# Validation of session ratings of perceived exertion for quantifying training load in karate kata sessions

**DOI:** 10.5114/biolsport.2022.109458

**Published:** 2021-10-25

**Authors:** Daniel Bok, Nika Jukić, Carl Foster

**Affiliations:** 1 Faculty of Kinesiology, University of Zagreb, Zagreb, Croatia; 2 University of Wisconsin-La Crosse, La Crosse, USA

**Keywords:** Martial arts, Heart rate, Internal load, Training impulse, Monitoring

## Abstract

This study was intended to investigate the associations between session Ratings of Perceived Exertion (sRPE) and Edwards’ training load (TL) and Banister training impulse (TRIMP) in order to determine the validity of the sRPE method for TL assessment in karate kata discipline. Eight elite karate kata athletes, members of the national karate team, took part in this study. A multistage 20 m shuttle run test was performed to determine maximal heart rate (HR_max_). Subsequently, the subjects performed 3 different kata training sessions separated by minimally 48 hours. To calculate Edwards TL, Banister TRIMP and sRPE, heart rate (HR) was continuously monitored during the sessions and RPE of the entire session was collected 30 minutes after each training session. The Pearson correlation coefficient (r) was used for determining associations between TL variables. Edwards TL (p = 0.064) and Banister TRIMP (p = 0.102) were not, but sRPE was significantly different between each training session (p < 0.001). There were no significant correlations between sRPE and Edwards TL (r = 0.53, p = 0.18) or Banister TRIMP (r = 0.13, p = 0.77) when data from all training sessions were pooled. A significant correlation was obtained between sRPE and Edwards TL (r = 0.71, p = 0.04) in situational training session, whereas in technical training session sRPE was significantly correlated with Banister TRIMP (r = 0.82, p = 0.01). HR-based methods for TL assessment are not able to discriminate between kata training sessions and, therefore, sRPE may be more useful for coaches to monitor TL in karate kata athletes.

## INTRODUCTION

Karate is one of the most popular combat sports in the world and was recently included into the program of the summer Olympic Games and is expecting its premiere in Tokyo 2021 [1]. The competition is organized in two disciplines, kumite and kata, which differ significantly in terms of performance characteristics and physiological demands [2]. Kata performance time is shorter with lower aerobic and greater anaerobic contribution to energy supply in comparison to kumite [2, 3]. Although it seems that physical profiles of kata and kumite athletes do not differ significantly [4], technical and tactical elements as well as the muscular activation and movement patterns of these two disciplines are highly different and require a high level of specialization of athletes from an early stage of their careers [3]. Namely, kata are predetermined sequences of karate techniques and are performed identically under situational conditions whereas the selection and frequency of punches and kicks in kumite are mostly influenced by the pressure of the opponent and the score line dynamic [5] in addition to the weight category and gender of the karateka [6]. Additionally, kumite are intermittent activities as the fights are interrupted by referees when points are gained whereas kata are continuous activities that offer no breaks until the very end. Accordingly, the contents and the structure of the training sessions as well as the training load imposed on athletes are greatly different in these two disciplines.

Monitoring athletes’ training load is essential for adjusting the optimal balance between stress and recovery, managing individual acute responses and adaptations to training programs and minimizing the risk of overtraining and injury [7]. One of the most commonly used methods for quantifying training load is the session Rating of Perceived Exertion (sRPE) [8] which has been shown to be a valid and reliable measure in a large number of sports and activities [9, 10]. As heart rate (HR) is the most commonly measured internal training load (TL) marker [11], the validity of the sRPE method had mostly been tested by assessing correlation coefficients with heart rate derived TL measures such as Edwards’ TL and Banister training impulse (TRIMP) [9]. Several recent studies confirmed that sRPE is also a valid measure of training load assessment during karate training sessions and competition matches [12]. Namely, significant correlations between sRPE and Edwards’ TL (r = 0.81) and Banister TRIMP (r = 0.79) were found for kumite technical training session [13]. Similarly, significant correlations between sRPE and Edwards’ TL (r = 0.79 and r = 0.80) and Banister TRIMP (r = 0.63 and r = 0.81) were found in two studies analyzing several different training sessions performed during a training camp [14, 15], whereas significant correlations of 0.77 and 0.84 between sRPE and Edwards’ TL and Banister TRIMP, respectively, were also found for competitive karate matches [16]. Although all reported studies confirmed validity of the sRPE method for assessing training load in karate they were all conducted on kumite athletes during similar specific training sessions and competition. Whether these significant correlations apply to different kata training sessions as well and whether sRPE can be used for assessing training load in the kata discipline is still not known. Therefore, the purpose of this study is to investigate the associations between sRPE and Edwards’ TL and Banister TRIMP in three different kata training sessions, which are often used in kata training, in order to determine the validity of the sRPE method for TL assessment in karate kata discipline. We hypothesized that sRPE would be a valid measure of TL in kata discipline.

## MATERIALS AND METHODS

### Subjects

Eight elite karate kata athletes, all members of the national karate team with several years of international competition experience, took part in this study. The subject sample consisted of four men (age 24.3 ± 6.5 years, height 179.8 ± 3.4 cm and body mass 84 ± 8 kg) and four women (age 20.3 ± 9.2 years, height 163.0 ± 6.2 cm and body mass 59.3 ± 3.2). All participants performed at least 7 karate training sessions with 2 additional strength and conditioning sessions per week and four of them won medals at the European championships in junior and senior categories within the last five years. The athletes and parents of the three minors who participated in the study were informed about the experimental procedures and provided written informed consent. The subjects were aware that they could withdraw from the study at any point without any consequences. The study protocol was approved by the Ethics Committee of the Faculty of Kinesiology University of Zagreb and conformed to the recommendations of the Declaration of Helsinki.

### Experimental procedure

At the beginning of a regular training session the participants performed a multistage 20 m shuttle run test for the purpose of determining maximal heart rate (HR_max_). Subsequently, they were required to perform three different kata training sessions separated minimally by 48 hours but scheduled at the same time of the day. The participant rested quietly for five minutes before each training session to record the lowest HR, which was considered as the resting HR. The first training session included foam rolling, running interspersed with calisthenics and flexibility exercises as warm-up, random short kata sequences performed in preferential order and volume and, for the main part of the session, four kata performances with 10 minutes active breaks between each. This training session simulated real competition and, therefore, was considered a situational training session. The second training session included foam rolling, running drills with speed and agility footwork and low-intensity plyometrics as warm-up, dynamic stretching followed by practice of basic karate techniques and, for the main part of the session, execution of three kata performances divided in half with 3 minutes passive breaks between halves and 15 minutes active breaks between each kata. This training session is often used by kata athletes to simultaneously improve their technical performance and for specific conditioning and is, therefore, considered a specific training session. Dividing kata in two similar halves decreases the overall physiological load and enables athletes to focus on technical proficiency, especially in the second half of the kata. The third training session included foam rolling, dynamic stretching and ball play as warm-up and, for the main part of the session, execution of three different kata divided in six short technical sequences each, with short random breaks between each sequence. The main goal of this training session is to improve technical performance by paying attention to details and, therefore, this session is considered a technical training session. The main part of each training session, in which kata performances were executed, needed to be performed with full intensity simulating competition conditions. The same katas were performed in all three training sessions in order to offset the possible influence of particular kata’s difficulty on sRPE or HR responses. The mean duration of the sessions was 86.7 ± 4.7 minutes, therefore, minimizing the possible influence of the session duration on the study results. Heart rate was continuously monitored throughout the entire session. Thirty minutes after each training session the participants provided their sRPE using the modified CR-10 scale [8, 10]. All participants were familiar with the RPE scale as they had used it for several weeks prior to this study. The menstrual cycle phase of the female participants was not controlled as we believed that it would not affect the results of this study even if trivial reduction in certain fitness components would occur in some individuals [17].

### Fitness testing

For the purpose of assessing the HR_max_ of each participant, a multistage 20-m shuttle run test was performed [18]. To complete the test the participants had to run back and forth between two lines 20 m apart following the pace of the prerecorded audio signal. Starting speed of the test was 8.5 km/h and running speed was increased by 0.5 km/h each minute. The test was stopped when the participant was no longer able to cover the distance in the required time and the highest HR attained at exhaustion was considered as HR_max_. Heart rate was recorded with Polar Team System (Polar, Kempele, Finland) HR monitor.

### Training load quantification

Training load was calculated using the sRPE method, Edwards’ TL and Banister TRIMP. Session RPE TL was calculated by multiplying RPE score with training session duration, expressed in minutes [8, 10]. Edwards’ TL was calculated by summing the results of time spent in each of the five HR zones expressed in minutes multiplied by a corresponding coefficient (50–60% HR_max_ × 1, 60–70% HR_max_ × 2, 70–80% HR_max_ × 3, 80–90% HR_max_ × 4, 90–100% HR_max_ × 5) [19], whereas Banister TRIMP was calculated through the formula:


$$\begin{array}{l} \text{Session duration}\times ({\text{HR}}_{\text{ex}}{\text{-HR}}_{\text{rest}})/({\text{HR}}_{\text{max}}{\text{-HR}}_{\text{rest}})\times 0.64{\text{e}}^{1.92\text{x}}\\ \text{Session duration}\times ({\text{HR}}_{\text{ex}}{\text{-HR}}_{\text{rest}})/({\text{HR}}_{\text{max}}{\text{-HR}}_{\text{rest}})\times 0.86{\text{e}}^{1.67\text{x}} \end{array}$$

for men and women, respectively, where e = 2.712 and x = (HR_ex_-HR_rest_)/(HR_max_-HR_rest_) [20]. In the equations HR_ex_ is the average HR during exercise, HR_rest_ is the resting HR and HR_max_ is the maximal HR.

### Statistical analysis

Data are presented as mean ± standard deviation (SD). Normality assumptions were verified using the Kolmogorov-Smirnov test. Oneway analysis of variance with repeated measurements and Tukey *post hoc* test were used for testing statistical significance in TL between training sessions. Pearson correlation coefficients (r) were used for determining associations between training load variables. The magnitude of correlation coefficients was considered trivial (r < 0.1), small (0.1 < r < 0.3), moderate (0.3 < r < 0.5), large (0.5 < r < 0.7), very large (0.7 < r < 0.9) and almost perfect (r > 0.9) [21]. Statistical significance was accepted at p < 0.05. Statistical analyses were performed with Statistica (v 13.2; Dell Inc, Tulsa, OK, USA).

## RESULTS

**FIG. 1:**
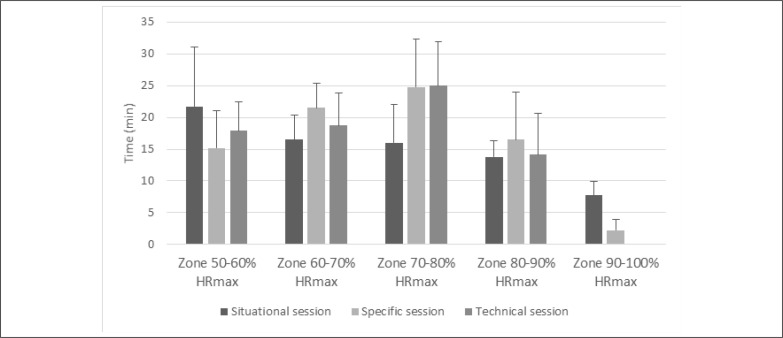
Average time (with standard deviations presented in bars) spent in different heart rate intensity zones for situational, specific and technical training session.

**FIG. 2:**
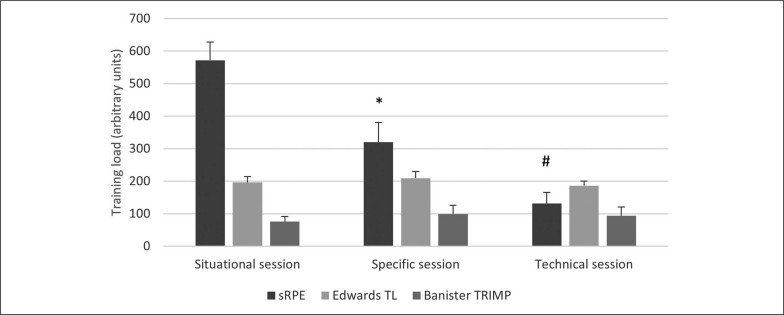
Training load of the three training sessions assessed through sRPE, Edwards TL and Banister TRIMP. *Significantly different from situational session (p<0.001) and technical session (p<0.001); #Significantly different from situational session (p<0.001) and specific session (p<0.001).

Distance covered during the multistage 20-m shuttle run test was 1557.5 ± 317.2 m. Peak HR during situational, specific, and technical training session were 183.5 ± 4.1 bpm (97.3 ± 2.1% HR_max_), 174.3 ± 5.9 bpm (92.4 ± 2.5% HR_max_), and 159.4 ± 12.5 bpm (84.5 ± 5.4% HR_max_), respectively. Time spent in different HR intensity zones for each training session is presented in Figure 1. Training loads of the three training sessions were not significantly different when expressed in Edwards TL (p = 0.064) and Banister TRIMP (p = 0.102), but showed significant difference when expressed through sRPE (p < 0.001) (Figure 2).

Pearson correlation coefficients between TL variables on pooled data and data separated on three training sessions are presented in Table 1.

**TABLE 1:** Pearson correlation coefficients between sRPE, Edwards TL and Banister TRIMP during situational, specific and technical training session and on pooled data

	Edwards TL	Banister TRIMP
**sRPE (pooled data)**	r = .53, p = .18	r = .13, p = .77
**sRPE (situational TS)**	r = .71, p = .04	r = .39, p = .33
**sRPE (specific TS)**	r = .52, p = .19	r = .01, p = .98
**sRPE (technical TS)**	r = .22, p = .60	r = .82, p = .01

Legend: sRPE – session ratings of perceived exertion, TS – training session.

## DISCUSSION

The main findings of this study were that 1) contrary to sRPE, the HR-based methods for assessing TLs cannot discriminate between different kata training sessions, 2) sRPE is not associated with heart rate-based methods when data from all sessions were pooled, 3) sRPE is largely correlated with Edwards TL during situational training session, 4) sRPE was not significantly correlated with any of the HR-based methods during the specific training session, and 5) sRPE was largely correlated with Banister TRIMP during the technical training session.

Training loads of the three training sessions appear to be the same when they were analyzed with HR-based methods. However, sRPE method was able to discriminate between them as all three training sessions were significantly different from each other. These particular training sessions were chosen for analysis as they were believed to represent the most commonly used variants of kata training sessions. In all training sessions the athletes were required to execute the same kata performances with full intensity, however, the durations of the performances were different across sessions. Namely, four whole kata performances were executed in the situational training session, six halves of three different katas were executed in the specific training session and 18 short kata sequences were executed in the technical training session. Duration of a kata performance is usually between 130 and 160 seconds [2] so kata halves could last between 60 and 80 seconds, whereas short kata sequences last for 10 to 20 seconds. Even though the overall work across the training sessions was quite similar, the different work and recovery distributions yielded a different internal physiological load. Whole kata performances, lasting approximately 2 minutes, induced the highest HR responses among athletes with peak HR values being 183.5 ± 4.1 bpm or 97.3 ± 2.1% HR_max_, but this high cardiovascular strain contributed little in terms of the entire training session. Performing kata halves also induced relatively high HR responses with peak HR values being 174.3 ± 5.9 bpm or 92.4 ± 2.5% HR_max_, but this cardiovascular strain during specific training session was even shorter. These short spikes in HR did not make a great difference on training load assessed through HR-based methods as the training sessions were rather long. Therefore, Edwards TL and the Banister TRIMP were not different across the training sessions leading to the conclusion that these three training sessions induced identical training stress to athletes. However, two-minute high-intensity exertions can cause large metabolic stress with blood lactate concentrations > 10 mmol/l [3, 22], whereas performing kata halves could also cause considerable blood lactate accumulation as they present 60 to 80-second exertions. The possible differences in metabolic stress induced during training sessions could be the reason of significantly different sRPEs. Indeed, very large correlations (r = 0.91) between sRPE and lactate-based TRIMP [13] and between sRPE and blood lactate concentration (r = 0.96) [23] were noticed for karate training sessions comprised of intensive 2-minute kumite bouts separated with 30 to 60 seconds recovery periods. Even in small-sided soccer games, which have lower metabolic and higher cardiovascular overall stress than kata performances, blood lactate data explained an additional 14.7% of RPE variance above the 43.1% that was explained with HR data alone [24]. While these studies show that metabolic stress is an important contributor to the sRPE, the results of this study indicate that for kata training sessions in particular its influence is much bigger than the influence of cardiovascular stress. That is probably the main reason why no meaningful correlations between sRPE and HR-based methods were found when data from all sessions were pooled together. Therefore, even though sRPE did not show significant associations with training load assessed through HR-based methods, which are commonly used for its validation, sRPE appears to have good ecological validity and presents a better solution when assessing TL in kata training.

During situational training session sRPE showed significant association with Edwards TL and no association with Banister TRIMP. This is somewhat different to the previously conducted studies on karate athletes as in all those studies significant and similar correlations were found between sRPE and both Edwards TL and Banister TRIMP during training [13, 14, 15] and competition [16]. However, all those studies were conducted on kumite athletes as they performed simulated or official matches. Kumite activities are longer in duration and utilize more aerobic energy whereas top-level kata performances rely more on the anaerobic energy contribution [25, 26]. It has recently been reported that during official kumite matches athletes spend 65% [27] to 74% [16] of total match time exercising in the HR zone > 90% HR_max_. Greater blood lactate accumulation during kata performance was also observed as similar concentrations were obtained after kata and kumite even though kata is usually about half the duration of a kumite bout [2, 3]. Additionally, sRPE after simulated (4.9 ± 0.6) [28] and official (4.2 ± 1.2) [16] kumite matches are rather low, whereas it was much higher (7.6 ± 0.7) after situational kata training session performed in this study, suggesting higher metabolic stress [10]. Therefore, the larger and significant correlations between sRPE and both HR-based methods for TL assessment that were found in kumite studies are not surprising. On the other hand, different correlations between sRPE and Edwards TL and Banister TRIMP found for this study can be explained with differences in the index calculation between two HR-based methods. Whereas both methods are limited to assess TL of intermittent exercises, Edwards TL is modified to facilitate the quantification of interval training by multiplying time spent in higher HR zones with a higher factor [29]. This makes it more sensitive to short bursts of high-intensity activities that elicit high HR. So, even though the Edwards TL underestimates TL when athletes spent more time in low-intensity HR zone [29], as is the case in this session, it still has the ability to discriminate athletes according to TL, which enabled significant correlation to sRPE. Conversely, Banister TRIMP is based on the extent to which activity raises HR in relation to resting and maximal HR, therefore, it is more influenced by mean HR during the session [29]. This is also supported by the fact that a significant correlation (r = 0.78) between Banister TRIMP and mean HR during situational session was found for this study. Because this training session is comprised of short burst of high-intensity activities and very long recovery periods rendering random and similar mean HR among athletes, it could not be used as appropriate indicator of TL. Therefore, Banister TRIMP was unable to distinguish TL differences between athletes and was only moderately correlated with sRPE.

It was interesting to find that there were no significant correlations between sRPE and either of the HR-based methods obtained during specific training session, even though the correlation between sRPE and Edwards TL appeared to be large. Specific training session included execution of three katas divided in halves with fairly long and passive between-halves and between-kata breaks. As kata halves usually last for 60 seconds both the aerobic and anaerobic contribution to performance is high, but much lower than during the situational training session. It was previously shown that blood lactate accumulation increases as a function of kata duration reaching a more than 3 mmol/l increase over resting state after an 80-second kata performance [30]. Extending kata performance from 30 to 60 seconds also generated 2.4 mmol/l higher blood lactate concentration as it increased from 4.5 mmol/l to 6.9 mmol/l [26]. Additionally, aerobic contribution doubled [26] and quadrupled [30] when kata performance was extended from 30 to 60 seconds and from 10 to 80 seconds, respectively. Therefore, it seems clear that performing kata halves could possibly induce moderate to high metabolic stress as indirectly estimated from the average sRPE (4.0 ± 0.8) and subsequent sRPE TL results obtained in the study. However, due to the rather long recovery periods, this perceptually moderate-to-high TL was not reflected in HR-based methods. In any case, the reason for low and non-significant correlations between sRPE and HR-based methods might also partially be related to the athlete’s poor ability to use RPE which might be caused by young age and training session characteristics [31]. Our subjects’ sample did include minors who might have exhibited poorer RPE skills, especially during such sessions in which short high-intensity activities are interspersed with long recovery periods making it harder to pinpoint actual perceptual exertion 30 minutes after the session. However, given the relatively large experience with sRPE in these athletes, we think that lack of familiarity or skill with rating is unlikely. It is also known that the correlation between HR and RPE in adolescents is less pronounced than in adults [31] and this could also be a small contributing factor for lower association between sRPE and HR-based methods, especially within the specific training session in which frequent changes of low and very high HRs were the main characteristic of the session. Additionally, due to its specific upper-extremity explosive movements and isometric lower-extremity positions karate exhibits much higher HR for a given %VO_2max_ [32] which also might contribute to the mismatch between physiological and perceptual load. Finally, apparent differences in fitness status evident through the results of the multistage 20-m shuttle run test, probably further contributed to the mismatch.

Conversely to the results obtained for the situational training session, during technical training session sRPE showed significant correlation with Banister TRIMP and no association with Edwards TL. This training session included performance of short kata sequences interspersed with random rest periods long enough for full recovery. Such short sequences usually last less then 15 seconds and are not very aerobically taxing [30]. This is supported by very low sRPE (1.8 ± 0.5) obtained after the session and the fact that there was no time spent in the > 90% HR_max_ zone. Because of that Edwards TL method got a bit less fit to discriminate between athletes whereas Banister TRIMP, which is more dependent on the mean HR, was better able to capture the small between-athlete differences in TL during this session. This is supported by the fact that almost perfect correlation (r = 0.99) was found between mean HR and Banister TRIMP in this session.

The fact that blood lactate concentration was not measured after the session, which would enable testing its association with sRPE, could be considered a study limitation. Additionally, a long warm-up, which was almost identical in all training sessions, could also have contributed to poor correlations between sRPE and HR-derived TL indexes and should be investigated in future studies. As only one session per session type was investigated, we were also unable to report the reliability of the sRPE at this point. Therefore, future studies should assess the reliability of the sRPE and use other types of kata training sessions, especially the situational ones comprised of different number of kata performances, to verify the results of this study. And finally, notwithstanding the fact that homogenous group of top-level kata athletes is hard to find, future studies should aim to recruit more athletes for their analysis in order to reach higher level of generalization power.

The results of this study show that sRPE may be a better index of karate kata session TL than HR-derived methods due to its better ecological validity. Coaches are, therefore, advised to use sRPE as a primary method of TL assessment during kata training sessions. Due to the fact that kata performances are rather short and metabolically highly demanding, HR-based methods might not be able to capture the real TL during kata training sessions. Therefore, HR monitoring should be used complementary to sRPE and only to capture the cardiovascular stress of the session.

## CONCLUSIONS

The results of this study show that HR-based methods for TL assessment are not significantly correlated with sRPE when data of all three sessions are pooled together. Edwards TL and Banister TRIMP are not able to discriminate between different kata training sessions whereas sRPE presents good ecological validity. Therefore, sRPE should be used preferably for TL assessment in kata training.

## Financial support

There was no grant funding received for this research.

## Technical assistance

No technical assistance was provided outside the list of authors.

## Intellectual contributions

There was no intellectual input from persons outside the list of authors.

## Conflict of interest statement

The authors of the manuscript do not have any conflict of interest to report.
